# High-Resolution Magic Angle Spinning Nuclear Magnetic Resonance Spectroscopy of Paired Clinical Liver Tissue Samples from Hepatocellular Cancer and Surrounding Region

**DOI:** 10.3390/ijms25168924

**Published:** 2024-08-16

**Authors:** Wendy M. Fernandes, Nicola Harris, Ane Zamalloa, Lissette Adofina, Parthi Srinivasan, Krishna Menon, Nigel Heaton, Rosa Miquel, Yoh Zen, Geoff Kelly, James A. Jarvis, Alain Oregioni, Shilpa Chokshi, Antonio Riva, I. Jane Cox

**Affiliations:** 1 The Roger Williams Institute of Hepatology, Foundation for Liver Research, 111 Coldharbour Lane, London SE5 9NT, UKa.riva@researchinliver.org.uk (A.R.); 2 Faculty of Life Sciences & Medicine, King’s College London, London WC2R 2LS, UK; 3 Institute of Liver Studies, King’s College Hospital NHS Foundation Trust, Denmark Hill, London SE5 9RS, UK; 4 Liver Histopathology Laboratory, Institute of Liver Studies, King’s College Hospital NHS Foundation Trust, Denmark Hill, London SE5 9RS, UK; 5 MRC Biomedical NMR Centre, The Francis Crick Institute, 1 Midland Road, London NW1 1AT, UK; 6 Randall Centre for Cell & Molecular Biophysics and Centre for Biomolecular Spectroscopy, King’s College London, London SE1 1UL, UK

**Keywords:** hepatocellular carcinoma, NMR spectroscopy, lipid metabolism, bacterial metabolites, personalised treatment

## Abstract

The global burden of liver cancer is increasing. Timely diagnosis is important for optimising the limited available treatment options. Understanding the metabolic consequences of hepatocellular carcinoma (HCC) may lead to more effective treatment options. We aimed to document metabolite differences between HCC and matched surrounding tissues of varying aetiology, obtained at the time of liver resection, and to interpret metabolite changes with clinical findings. High-resolution magic angle spinning nuclear magnetic resonance (HRMAS-NMR) spectroscopy analyses of N = 10 paired HCC and surrounding non-tumour liver tissue samples were undertaken. There were marked HRMAS-NMR differences in lipid levels in HCC tissue compared to matched surrounding tissue and more subtle changes in low-molecular-weight metabolites, particularly when adjusting for patient-specific variability. Differences in lipid-CH_3_, lipid-CH_2_, formate, and acetate levels were of particular interest. The obvious differences in lipid content highlight the intricate interplay between metabolic adaptations and cancer cell survival in the complex microenvironment of liver cancer. Differences in formate and acetate might relate to bacterial metabolites. Therefore, documentation of metabolites in HCC tissue according to histology findings in patients is of interest for personalised medicine approaches and for tailoring targeted treatment strategies.

## 1. Introduction

Hepatocellular carcinoma (HCC) is a primary malignancy and an end-stage chronic liver disease (CLD) which accounts for 75–85% of all liver cancers. CLD, especially cirrhosis resulting from alcohol abuse, metabolic dysfunction-associated steatotic liver disease (MASLD), hepatitis B virus (HBV) and hepatitis C virus (HCV) infections, substantially elevates the risk of hepatocellular carcinoma (HCC) development [1,2].

The need to improve early diagnosis, curative treatments, and support in regions with higher incidence is emphasized by the fact that HCC burdens the global healthcare systems by ranking as the third most lethal cancer after colorectal and lung cancers, with a 5-year survival rate of less than 20% in advanced stages [3]. In 2020, over 900,000 people were diagnosed with HCC and approximately 800,000 died from the disease, with mortality rates 2.2-fold higher in males compared to females [4]. The highest incidence of HCC mortality has been observed in Africa and East Asia, whereas the United States of America (USA) has seen a rapid increase in reported cancer deaths since the 2000s [5]. The incidence of HCC in England during 2010–2016 varied significantly by region, with higher rates in the North of England and London [6].

Advanced stages of chronic liver disease are thought to be driven by immune dysregulation, gut dysbiosis and metabolic perturbation [7,8,9]. During cirrhosis, a precursor stage to a proportion of liver cancer, damage to the intestinal lining correlates with local and systemic inflammation [10,11], and intestinal alterations have also been linked with immune impairment during advanced liver disease [12,13]. Dysregulation of fatty acid metabolism is a hallmark of cancer cells, and this is also the case in HCC development and progression [14]. Metabolic pathways affecting the production of lipids, particularly cholesterol and short-chain fatty acids, may be relevant for liver homeostasis and immune modulation [12,13]. Understanding lipid metabolic features of HCC could therefore lead to more effective treatment options, including optimising the immune response [15,16]. Furthermore, gut microbiota have been linked to treatment response to immunotherapy in melanoma and epithelial tumours [17,18,19,20], and a recent study showed that microbiota in the tumour itself may contribute to the pathology of HCC [21].

High-resolution magic angle spinning nuclear magnetic resonance (HRMAS-NMR) spectroscopy studies allow the analysis of the metabolic profile of intact tissue, without the need for tissue extraction and the associated limitations of the extraction methodology [22,23,24]. A range of liver tissue HRMAS-NMR studies have been reported, including liver biopsy assessment of donor liver and evaluation of donor liver graft function and donor-recipient matching [25,26]; the metabolic profiles associated with chronic hepatitis and cirrhosis with a range of underlying aetiologies [27], HCC [28,29,30], liver metastases [31,32,33,34], hepatoblastoma [34,35] and HCV [36]. Hepatic lipid tissue levels measured by HRMAS-NMR have been used to validate other novel spectroscopy techniques, for example, interventional fibre optic spectroscopy [37].

Previous HRMAS-NMR studies of HCC illustrated that the metabolic profile of HCC was influenced by tumour size, underlying aetiology, oncogenesis, and tumour grade [28,29,30]. However, HCC is considered one of the most heterogeneous tumour types [38], with variability not only between patients but also between and within tumours. Therefore, providing as much information as possible about the clinical characteristics of HCC and specific tumour biology will be relevant when developing new treatment options on a subject-specific basis [39]. We used HRMAS-NMR to generate subject-specific information for the metabolic characterisation of HCC tissues which have been used in our Institute for ex vivo models [39]. We aimed to document metabolite differences between liver tissue from HCC and the surrounding liver, obtained at the time of liver resection, and to correlate metabolite changes with clinical findings to assess the value of MAS NMR for additional metabolic characterisation of patient-specific tumours [40].

## 2. Results

The study cohort comprised N = 5 male, and N = 5 female subjects aged 70 (65, 79) years [median (Q1, Q3)] (Table 1). Four patients (patient IDs 116, 134, 141, and 168) had a confirmed diagnosis of steatotic liver disease (SLD) and four were reported to be regularly consuming alcohol (patient IDs 99, 117, 134, and 168).

The median (Q1, Q3) mass of the tissue sample loaded into the disposable insert was 16.7 (16.0, 18.0) mg. The median (Q1, Q3) 360o pulse length was 22.6 (22.6, 22.7) μs. The median (Q1, Q3) water linewidth measured from the pulse-collect NMR spectrum was 9.6 (8.9, 11.0) Hz.

Histological assessment of the liver tissue samples used for HRMAS-NMR analysis did not prove to be possible, as the tissue integrity was damaged by a combination of high-speed spinning and removal from the disposable insert; therefore, H&E staining and histology assessment were carried out on archived and adjacent tissue samples from the same patient.

**Figure 1:**
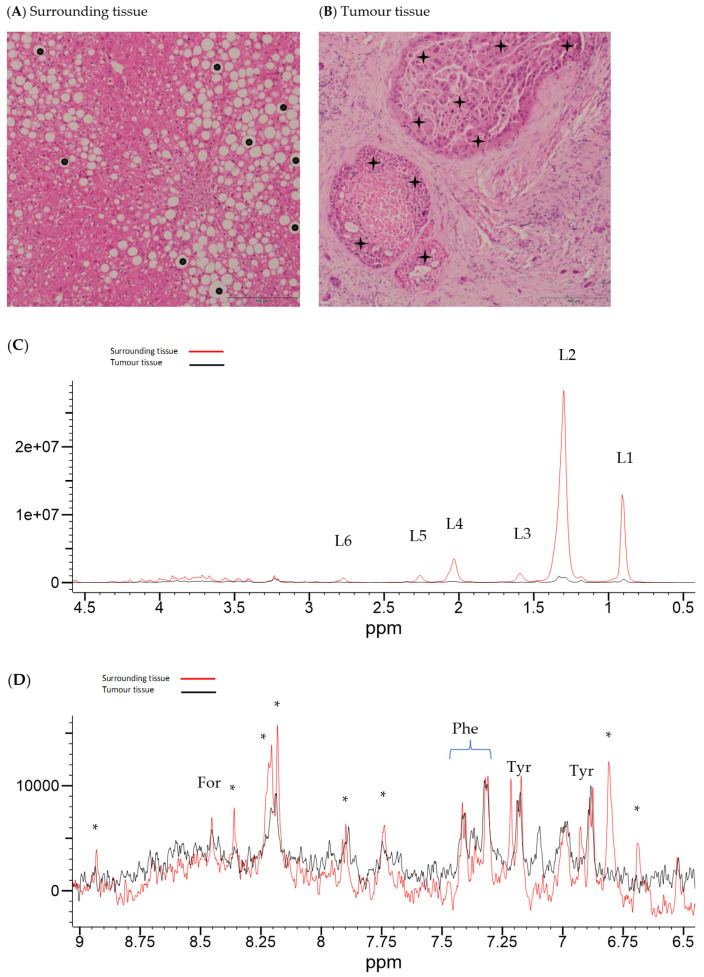
Histology and HRMAS-NMR data from surrounding tissue and HCC tissue from subject 039. Histology from surrounding liver tissue (**A**) shows moderate macrovesicular steatosis (added black dots), focal fibrosis, and mild inflammation. Histology for HCC (**B**) shows cancer cells (added black stars) arranged in nests against the background of the fibrotic stroma of subject 039 (underlying aetiology: alcohol-related liver disease, currently alcohol abstinent, previous cigarette smoker). Corresponding HRMAS-NMR spectra from surrounding (red line spectrum) and HCC tissue (black line spectrum) (sample ID 039A and 039B, respectively), illustrating the aliphatic region (**C**) and aromatic region (**D**), plotted on the same vertical scale within each spectral expansion. The aromatic region (**D**) is vertically expanded by approximately 1000× compared to the aliphatic region (**C**). NMR peaks were labelled in the expanded regions to L6, (lipid-CH=CHC**H_2_**-CH=CH- (polyunsaturated fatty acid)); L5, lipid CH_2_C**H_2_**CO (α-methylene to carboxyl); L4, (lipid CH=CHC**H_2_**CH_2_); L3, lipid C**H_2_**CH_2_CO (methylene to L5); L2, (lipid-C**H_2_**-); L1, (lipid-C**H_3_**), For (formate), Phe (phenylalanine), Tyr (tyrosine), and (*) to be assigned.

**Figure 2:**
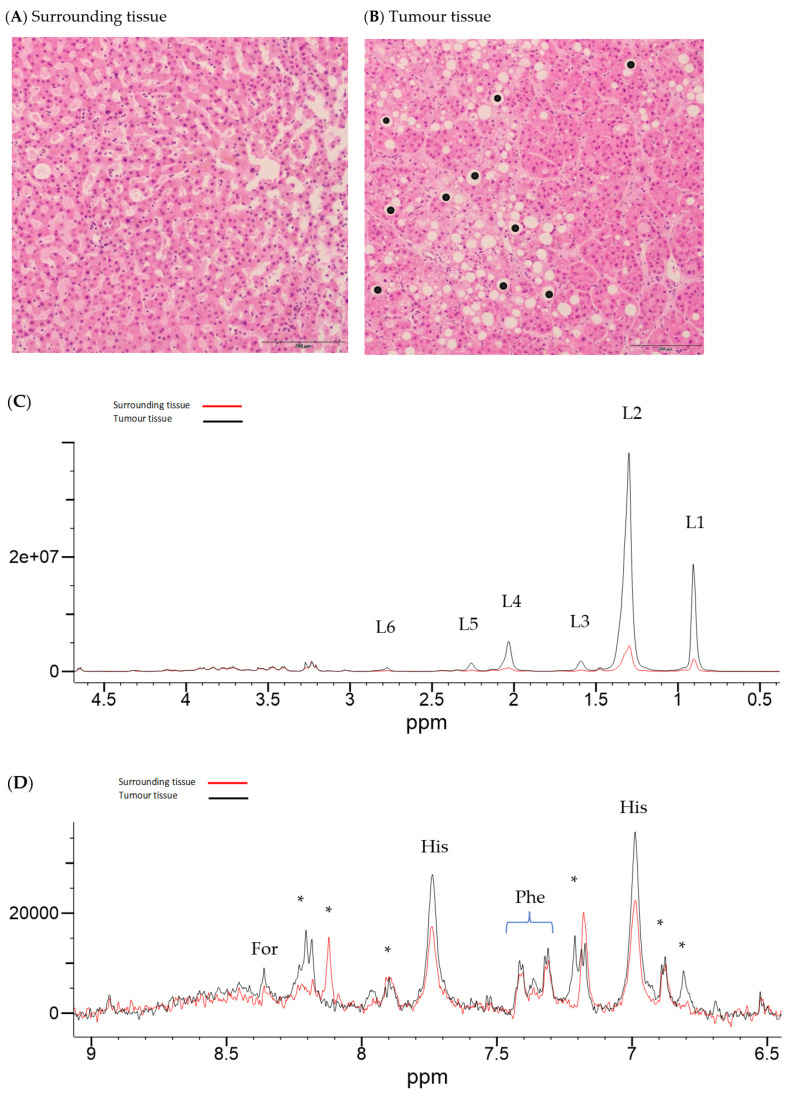
Histology and HRMAS-NMR data from surrounding tissue and HCC tissue from subject 117. Histology from surrounding liver tissue (**A**) shows only mild non-specific microscopic changes without the evidence of underlying chronic liver disease. The HCC (**B**) histology from subject 117 shows a moderately differentiated trabecular pattern of proliferation and moderate steatosis (added black dots), (underlying aetiology: unknown, current alcohol consumption 9 units/week, previous cigarette smoker). Corresponding HRMAS-NMR spectra from surrounding (red line spectrum) and HCC tissue (black line spectrum) (sample ID 117A and 117B, respectively), illustrating the aliphatic region (**C**) and aromatic region (**D**), plotted on the same vertical scale within each spectral expansion. The aromatic region (**D**) is vertically expanded by approximately 1000× compared to the aliphatic region (**C**). NMR peaks were labelled in the expanded regions to L6, (lipid-CH=CHC**H_2_**-CH=CH- (polyunsaturated fatty acid)); L5, lipid CH_2_C**H_2_**CO (α-methylene to carboxyl); L4, (lipid CH=CHC**H_2_**CH_2_); L3, lipid C**H_2_**CH_2_CO (methylene to L5); L2, (lipid-C**H_2_**-); L1, (lipid-C**H_3_**), For (formate), His (histidine), Phe (phenylalanine), and (*) to be assigned.

**Figure 3:**
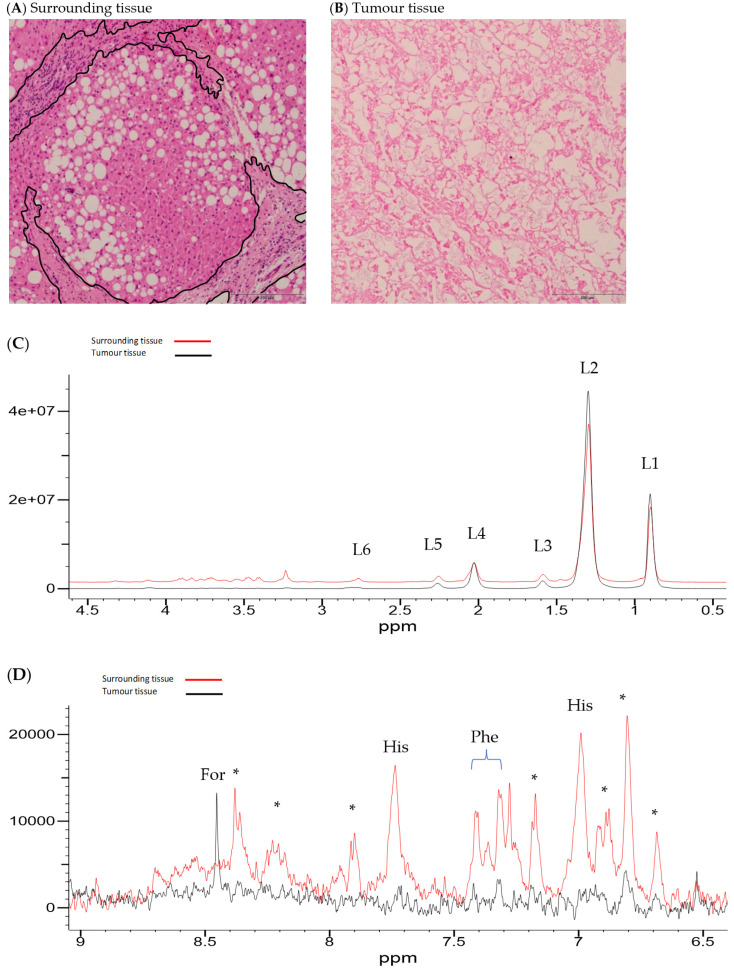
Histology and HRMAS-NMR data from surrounding tissue and HCC tissue from subject 134. Histology from surrounding liver tissue (**A**) shows a cirrhotic change, moderate steatosis, and mild inflammatory activity (regions marked in black). HCC (**B**) from subject 134 is necrotic (underlying aetiology: unknown, current alcohol consumption, 3.7 units/week, not a former cigarette smoker). Corresponding HRMAS-NMR spectra from surrounding (red line spectrum) and HCC tissue (black line spectrum) (sample ID 134A and 134B, respectively), illustrating the aliphatic region (**C**) and aromatic region (**D**), plotted on the same vertical scale within each spectral expansion. The aromatic region (**D**) is vertically expanded by approximately 1000× compared to the aliphatic region (**C**). NMR peaks were labelled in the expanded regions to L6, (lipid-CH=CHC**H_2_**-CH=CH- (polyunsaturated fatty acid)); L5, lipid CH_2_C**H_2_**CO (α-methylene to carboxyl); L4, (lipid CH=CHC**H_2_**CH_2_); L3, lipid C**H_2_**CH_2_CO (methylene to L5); L2, (lipid-C**H_2_**-); L1, (lipid-C**H_3_**), For (formate), His (histidine), Phe (phenylalanine), and (*) to be assigned.

**Figure 4:**
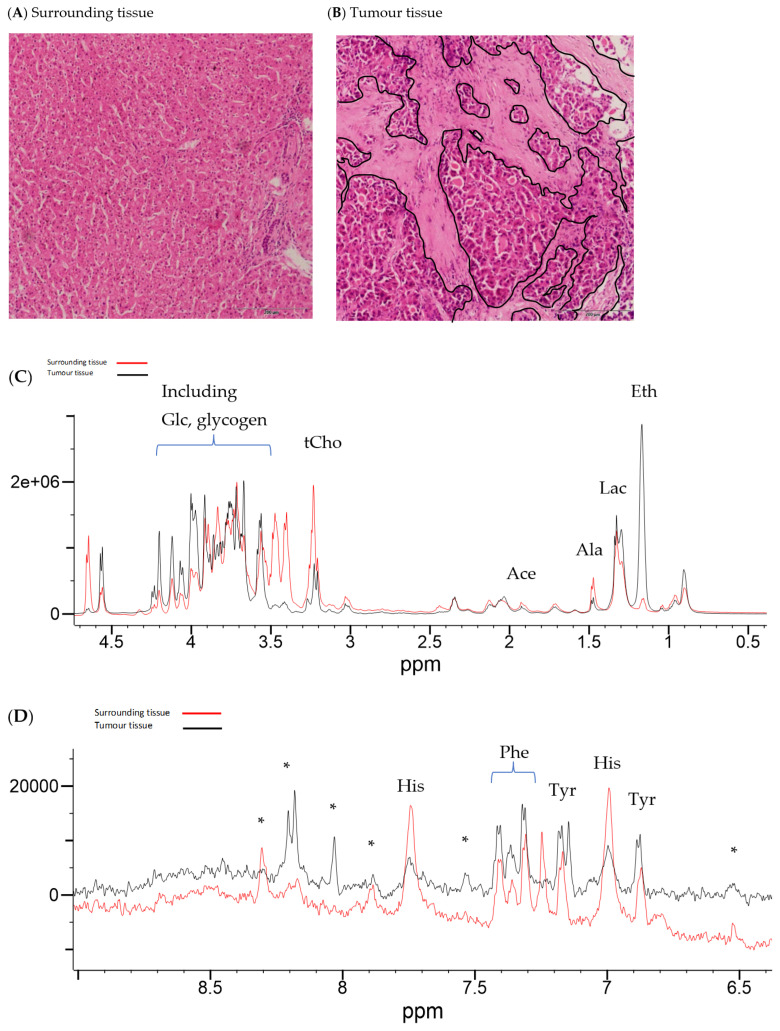
Histology and HRMAS-NMR data from surrounding tissue and HCC tissue from subject 099. Histology from surrounding liver tissue (**A**) shows mild periportal fibrosis with ductular reactions. HCC histology (**B**) from subject 099 shows cancer cells arranged in a trabecular architecture against the background of mildly fibrotic stroma (regions marked in black). (Underlying aetiology: CLD, alcohol consumption 10 units/week, smoker 10 cigarettes/day). Corresponding HRMAS-NMR spectra from surrounding (red line spectrum) and HCC tissue (black line spectrum) (sample ID 099A and 099B, respectively), illustrating the aliphatic region (**C**) and aromatic region (**D**), plotted on the same vertical scale within each spectral expansion. The aromatic region (**D**) is expanded by approximately 100× compared to the aliphatic region (**C**). NMR peaks were labelled in the expanded regions to Glc (glucose), tCho (choline-containing compounds), Ace (acetate), Ala (alanine), Lac (lactate), Eth (Ethanol), His (histidine), Phe (phenylalanine), Tyr (tyrosine), and (*) to be assigned.

**Figure 5:**
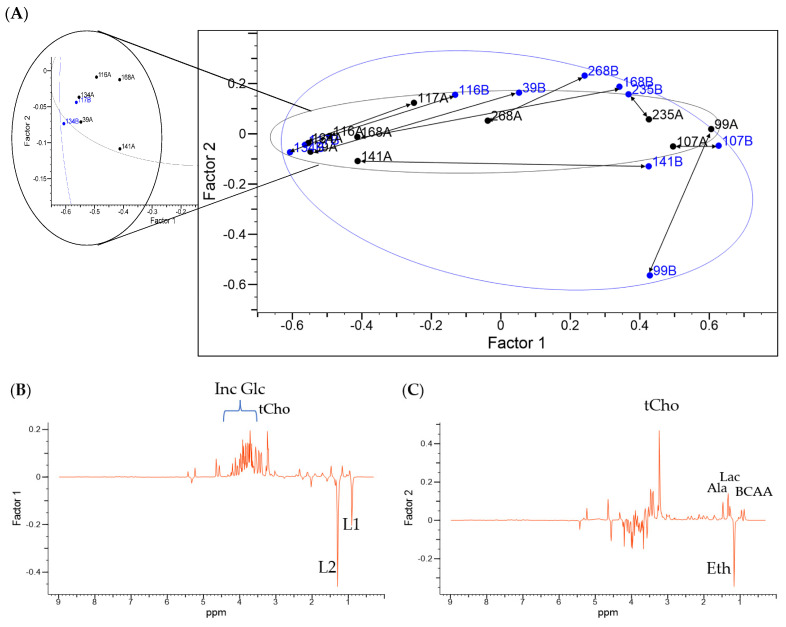
Multivariate analyses of NMR results from HCC and surrounding tissue. (**A**) Principal component analysis (PCA) plot with loading plots of (**B**) factor 1 and (**C**) factor 2 generated by the Wiley KnowItall software. Each loading plot represents the relative contribution of every original variable to the factor axis. NMR peaks were labelled in the expanded regions to L2, (lipid-C**H_2_**-); L1, (lipid-C**H_3_**), Glc (glucose), tCho (choline-containing compounds), Lac (lactate), Ala (alanine), Eth (ethanol), BCAA (branched-chain amino acids.

**Figure 6:**
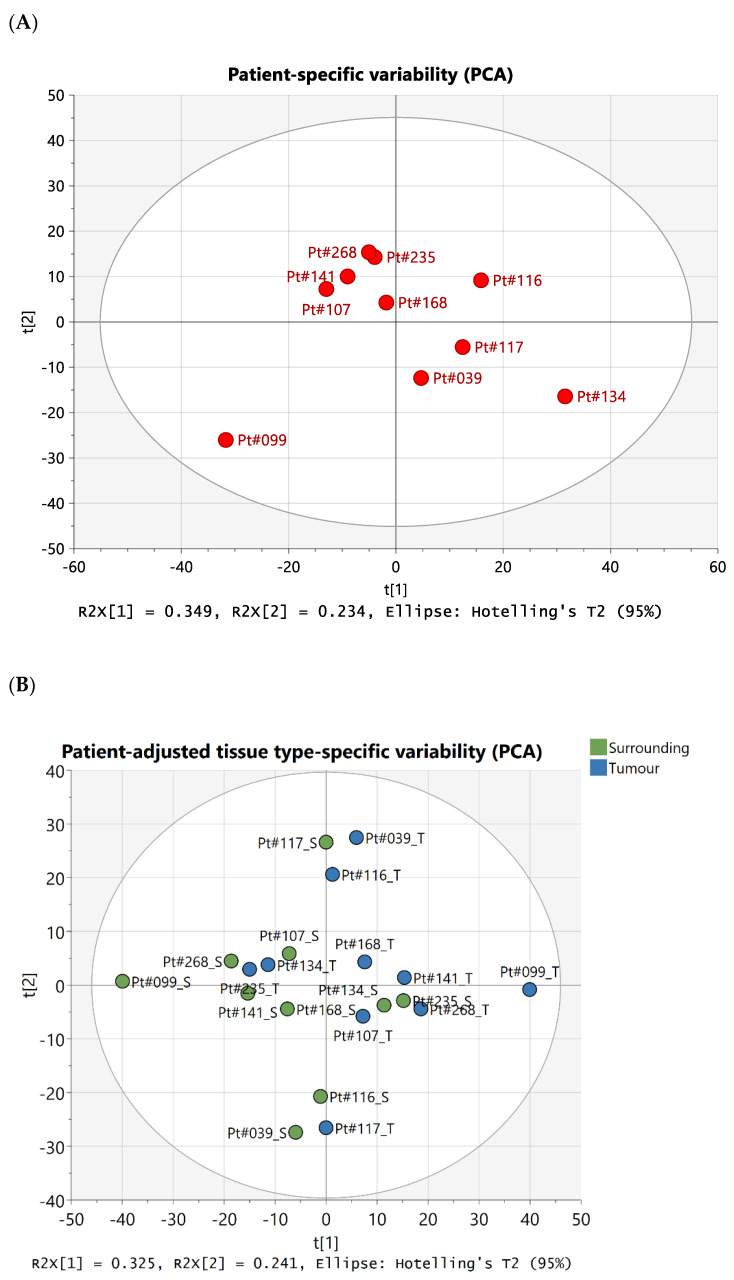
Multivariate analyses by source of variability. Principal component analyses (PCA) by (**A**) patient-averaged data, to investigate inter-subject variability, and (**B**) patient-adjusted data, to investigate variability due to matching of tissue type; each tissue sample in panel (**B**) is identified by the patient ID number and “S” for surrounding (green) or “T” for tumour (blue).

**Figure 7:**
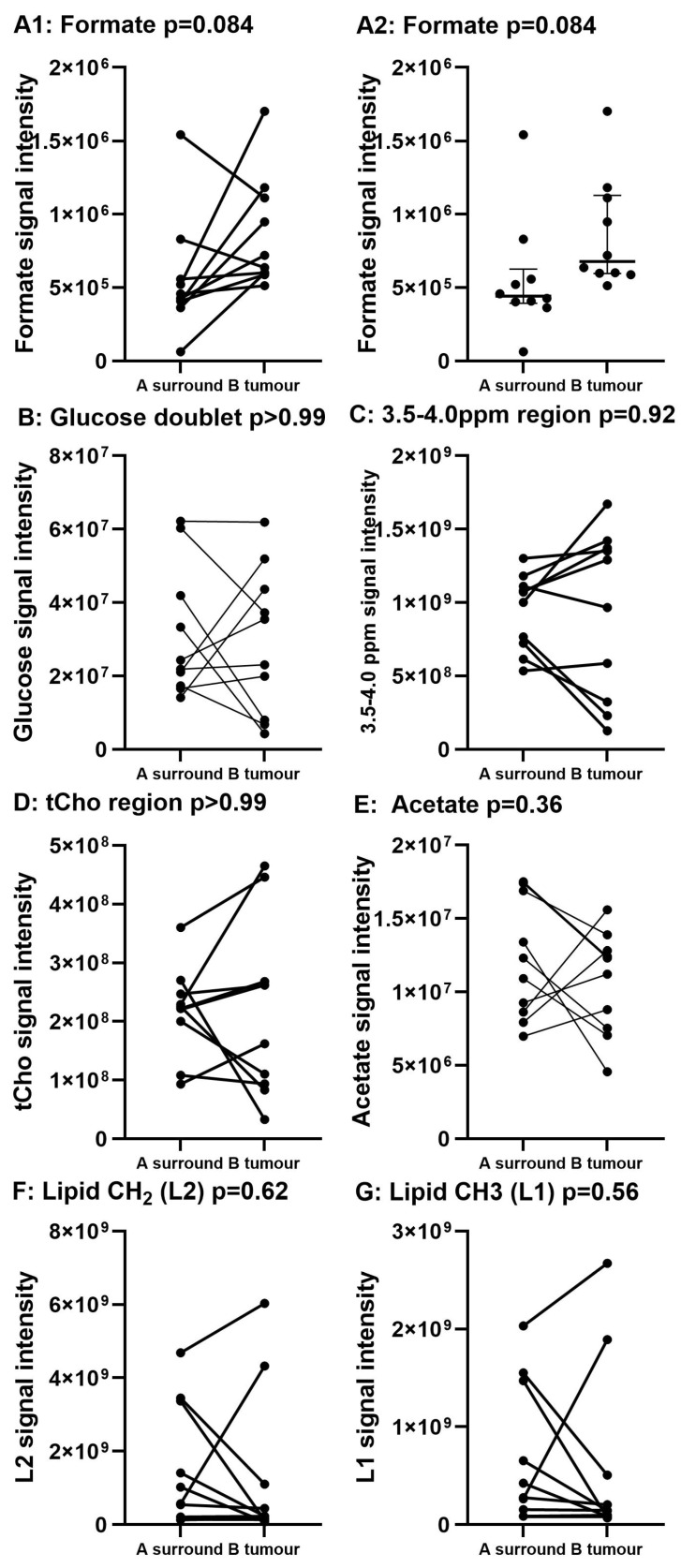
Relative signal levels of paired samples for specific metabolites. (**A1**,**A2**) formate, (**B**) glucose doublet, (**C**) 3.5–4.0 ppm region (**D**) region of choline-containing compounds (tCho), (**E**) acetate, (**F**) Lipid-CH_2_, (**G**) Lipid-CH_3_. *p* = values Wilcoxon matched-pairs signed-rank test. Differences in formate levels, between tumour and background liver tissues, were the only metabolite change that neared significance (*p* = 0.084).

Illustrative Carr–Purcell–Meiboom–Gill (CPMG) spin-echo HRMAS-NMR spectra from HCC and matched surrounding non-tumour liver tissue are summarised in Figure 1, Figure 2, Figure 3 and Figure 4 and compared to histological images archived from matched background liver tissue samples (peak assignments are listed in Supplementary Table S1). These NMR data sets show the heterogeneous range of metabolite differences between the paired tissue samples. Principal component analyses (PCA) of paired data points are illustrated in Figure 5 and Figure 6. Paired metabolite signal levels of specific metabolites (formate, glucose, total choline region, acetate and lipids), unadjusted for the mass of tissue loaded in the disposable insert, are illustrated in Figure 7 and summarised in Supplementary Table S2.

### 2.1. Metabolites Are Heterogeneous in HCC Tumour and Surrounding Tissue

There was considerable variability in the HRMAS-NMR metabolite and lipid levels (Figure 1, Figure 2, Figure 3, Figure 4, Figure 5, Figure 6 and Figure 7, Supplementary Table S2) in both HCC tumour and surrounding tissue samples. NMR lipid levels were of similar intensity in N = 4 HCC tumour tissue and matched surrounding tissue samples (patient IDs 99, 107, 134, and 235) and were clearly different for N = 6 subjects (patient IDs 039, 116, 117, 141, 168, and 268) (Figure 4A). Higher lipid signals tended to correlate with lower glucose levels and vice versa (PC1 loadings plot, percentage variance 73.7%, Figure 5B). The PC2 loading plot differences (percentage variance 10.6%, Figure 5C) were influenced by differential levels of choline-containing compounds and ethanol, most obviously illustrated by increased ethanol/reduced choline levels in sample ID 099B.

This initial analysis showed two main sources of variability: subject-specific variability coupled with differences between matched tumour and surrounding tissue samples. To separate and independently investigate these sources of variability, we performed unsupervised PCA analysis of patient-averaged data. The resulting broad spread and lack of clustering of individual patient points confirmed the strong individuality of liver tissue metabolic data (Figure 6A), and examination of spectral loadings indicated that varying combinations of lipids glucose (PC1; % explained variability 34.9%) and ethanol and metabolites in the aromatic region (PC2; % explained variability 23.4%) were indeed primarily responsible for subject-specific differences overall.

After correcting for subject-specific differences, analysis of patient-adjusted data showed that the residual paired differences between most tumour and surrounding samples (patient IDs 099, 107, 134, 141, 168, 235, and 268) related to spectral changes in the aromatic region (PC1; % explained variability 32.5%), whereas in a small subset of patients (patient IDs 039, 116, and 117), residual changes in lipids and glucose remained independently (orthogonally) dominant (PC2; % explained variability 24.1%), albeit comparable to loadings in the aromatic regions (Figure 6B).

### 2.2. Differences in Lipids Based on Aetiology

Mobile lipid levels in HCC tissue were highest in the two tumour samples identified either with high steatosis on histology (sample ID 117B) or as mostly necrotic (sample ID 134B).

The HRMAS-NMR spectra of three samples of surrounding tissue showed similar characteristics (surrounding tissues from patient IDs 099, 107, and 235 (Figure 5)), and these were from patients with either no underlying liver disease on histology, treated disease, or no steatosis identified by histology. Lipid levels in these subjects were similar in magnitude in the tumour tissue versus subject-matched surrounding tissue.

### 2.3. Type 2 Diabetes Mellitus

The patients with Type 2 diabetes mellitus (T2DM) show elevated glucose levels in tumour and surrounding tissue compared to individuals without T2DM. Three of four patients with underlying aetiology of SLD also had T2DM and were currently on Metformin (patient IDs 116, 134, and 168).

### 2.4. Formate

The spectral region 6.5–9.0 ppm, generally dominated by signals from aromatic compounds, tended to show fewer peaks in the tumour samples compared to samples from surrounding tissue. This was most obvious in the HRMAS-NMR spectrum from sample ID 134B, in which only one peak, assigned to the one-carbon molecule, formate, was observed. Indeed, an increase in relative formate levels in tumour tissue compared to surrounding tissue was the most consistent and nearly significant metabolite change overall (Wilcoxon signed-rank test, *p* = 0.084).

### 2.5. Ethanol

Ethanol peaks of varying intensity were detected in HCC tissue from five subjects (sample IDs 039B, 099B, 107B, 168B, and 235B) and in two paired tissue samples (sample IDs 099A and 235A). This was consistent with reported alcohol consumption by these subjects.

## 3. Discussion

A range of metabolites were detected in the N = 10 HCC tumour and surrounding tissue samples, with lipids and glucose generally being the dominant peaks. There was considerable variability in HRMAS-NMR lipid and metabolite levels (Figure 1, Figure 2, Figure 3, Figure 4, Figure 5, Figure 6 and Figure 7, Supplementary Table S2), in both HCC tumour and surrounding tissue samples. Lipid levels in tumour tissue were similar in magnitude to corresponding lipid levels in background liver tissue in four patients. Mobile lipid levels in HCC tissue were highest in the two tumour samples identified either with high steatosis on histology or as mostly necrotic.

In this study, histology was not possible on the tissue samples after HRMAS-NMR analyses. This is one aspect of the study that is important to address so that the metabolic profile can be more accurately correlated and interpreted with biopsy or tissue findings [41]. Several studies have considered the use of slow sample spinning [42,43,44], which may then allow combined HRMAS-NMR and histology of such liver tissue samples. However, at slower spinning rates, the overlap of spinning sidebands with relevant metabolites needs careful consideration [44], and this is likely to be a particular problem when lipid peaks dominate the HRMAS-NMR spectrum.

Our findings are consistent with the previous clinical HRMAS-NMR studies of HCC. For example, Yang et al. showed increases in a range of low molecular weight metabolites (including lactate, glutamate, and alanine) and reduced triglycerides, glucose, and glycogen in HCC samples from 17 patients with varying tumour grades compared to non-involved background liver tissue from 14 patients [28]. Solinas et al. compared HRMAS-NMR findings of tissue from needle biopsies (14 primary HCC nodules, 14 recurrent HCC, and 23 paired cirrhotic specimens) and showed metabolite differences between HCC and surrounding tissue (increased choline, TMAO, and decreased saturated fatty acids) and between recurrent and primary HCC (increased lactate and myo-inositol); they also reported decreased saturated fatty acids in large HCC nodules (>2 cm) [17]. Cao et al. considered the HRMAS-NMR profiles from HCC with differing underlying aetiology (N = 20 each for alcohol-related liver disease, HBV, and HCV infections) and showed how the metabolic phenotypes varied across aetiologies [30].

The finding of altered glucose and lipid metabolites in tumour tissue is consistent with the well-established Warburg effect, where cancer cells shift towards glycolysis, leading to increased anaerobic consumption of glucose [45]. This effect can also have a downstream impact on lipid metabolism. Lipid signalling plays a role in glucose metabolism by insulin regulation and glucose uptake, thereby affecting metabolic homeostasis [46,47]. Alteration in the metabolism of lipids such as phospholipids, sphingolipids, and prostaglandins can alter signalling pathways. A typical hallmark of type-2 diabetes is linked to the ability of certain lipids to impair insulin signalling, whereas increased lipogenesis could contribute to the de novo synthesis of fatty acids. Glucose levels (Table 1) were highest for patient ID 116 with untreated diabetes, whereas glucose levels were variable in patients with treated T2DM. The identification of lipid/glucose changes as dominant factors in patient-specific variations highlights the importance of metabolomic studies to identify personalised features that may aid diagnosis in these patients, whereas the identification of aromatic compounds and small metabolites as relevant factors beyond subject-specific variations for the discrimination of tumour and surrounding areas points at finer metabolic differences common to tumour or non-tumour cells, which warrant further investigations in this area.

Formate has been previously linked to carcinogenesis [48,49,50,51,52], and our data support this association. However, increased formate levels in tumour tissue could arise from differences in diet, differences in tumour-specific tissue metabolic alterations [53], or changes arising from altered gut microbiota metabolism [50]. The possibility of targeting formate metabolism could be explored as a potentially novel treatment strategy [48].

The higher levels of acetate observed in some of the tumour samples may arise from dietary differences, gut–liver axis changes, or metabolic processes within the tumour. Acetate is a key host metabolite with central roles in lipid biogenesis, which may link these findings to the already-described observations of predominant and differential lipid-related peaks [54].

Ethanol was detected in certain tissue samples, particularly evident in tumour tissue samples from subjects who disclosed a history of active alcohol consumption. Beyond the variation in alcohol intake, the increased ethanol level in some HCC tissue might also reflect different metabolic processes within the tumour or alterations of the gut–liver axis [55,56].

In a recent ex vivo precision cut tumour slice (PCTS) model study [39], we observed that patient ID 168 and 141 had differential checkpoint receptor expression in PCTS based on gene expression analysis, with patient 141 displaying a higher expression of programmed cell death protein 1 (PD-1) compared to patient 168, which may relate to an implicit difference in immune efficiency in the two subjects. The HRMAS-NMR data from tissue from patients 168 and 141 showed similar lipid levels in the surrounding tissue and lower lipids in the tumour, with the lowest levels in sample ID #168B. Formate levels were increased in the tumour tissue in both, whilst glucose was reduced.

The patients recruited to this study were selected as being suitable for liver resection, without preselection based on aetiology. Given the heterogeneity of NMR findings from patient to patient, questions to be addressed in further studies could include a study of NMR profiles according to aetiology, improved quantitation of metabolite levels by identifying an internal or external reference standard, whether HRMAS-NMR findings correlate with degree of differentiation, the metabolic impact of alcohol consumption or obesity on carcinogenesis, and how gene expression analysis relates to tissue metabolic profile.

## 4. Materials and Methods

Patients undergoing partial hepatectomy as a treatment for primary liver cancer were enrolled, and written informed consent was obtained for all the subjects involved in this study (Table 1 for clinical characteristics). This study was conducted according to the Declaration of Helsinki principles and approved by the local Research Ethics Committee established by the Health Research Authority (REC reference 17/NE/0340; IRAS project ID 222302; 31 October 2017). Samples were collected from October 2018 to February 2023. Patient recruitment included both males and females in an unbiased manner. The liver tissue was maintained at room temperature for 20 min before being snap-frozen in liquid nitrogen. The samples were then stored at −80 °C for 1–51 months before the NMR study.

Untargeted tissue NMR analysis of N = 10 paired HCC and surrounding liver tissue samples was undertaken. The clinical details of the patients are summarised in Table 1. Approximately 15 mg of liver tissue was carefully loaded into a Bruker 30 µL disposable insert, made from polychlorotrifluoroethylene (PCTFE), commonly known as Kel-F^®^, at the Roger Williams Institute of Hepatology. Then, 7 µL of D_2_0 was pipetted into the insert on top of the tissue before the insert was sealed with a plug and sealing screw. After filling and sealing, to ensure no residual external contaminants, the outside of the disposable insert was cleaned by wiping with 80% ethanol. The plugged and sealed Bruker HRMAS-NMR disposable inserts were transported at 4 °C in a UN3373-labelled cold bag by courier bike to the MRC Biomedical NMR Centre, The Francis Crick Institute, London.

NMR data collection was undertaken using standard NMR protocols using a Bruker Avance III 600 MHz system equipped with a Bruker 4 mm double resonance 1H, 13C HR-MAS probe (Bruker BioSpin GmbH, Ettlingen, Germany). Prior to the session for tissue HRMAS-NMR data acquisition, the probe temperature was calibrated using methanol and the shimming was optimised using a sample of sucrose (achieving a linewidth of 2 Hz for the added internal standard, sodium 3-(trimethylsilyl) propane-1-sulfonate).

Each disposable insert was visually checked before loading into the rotor (made from Zirconia) and capped with a finned cap made from PCTFE/Kel-F^®^. The fins on the cap were inspected under a stereomicroscope for any damage or dirt before the sample was loaded into the probe. The spin speed of the sample was gradually increased to 5000 Hz, to ensure stable spinning. The sample temperature was allowed to equilibrate to 4 °C before data collection was started. Additional shimming was carried out for each tissue sample, primarily by adjusting the Y-shim.

NMR spectra were obtained at 4 °C with a spinning speed of 5000 Hz. The 360° pulse length was confirmed for each sample, followed by a pulse-collect sequence (without water suppression, 90° pulse, fixed receiver gain 7.12, recycle delay 5 s, number of scans 8) and a Carr–Purcell–Meiboom–Gill (CPMG) spin-echo sequence (pre-saturation pulse of 2 s to suppress the large water signal, CPMG filter time 66 ms, fixed receiver gain of 256, recycle delay 3.1 s, number of scans 128). The time for NMR data acquisition was typically 30 min.

After NMR data acquisition, the samples were returned to the Roger Williams Institute of Hepatology to attempt subsequent histology and for clinical waste disposal. Histology was performed on both the HCC tumour and surrounding liver tissue to determine visual differences between both tissues.

### 4.1. NMR Spectral Processing and NMR Data Analyses

NMR spectra were processed (exponential line broadening 3 Hz) and phased using the Bruker software (TopSpin 4.0.7, Bruker BioSpin GmbH, Germany). All NMR spectra were referenced to the alanine peak at 1.48 ppm; no additional alignment of the various NMR spectral peaks was required. Prior to further analyses, the residual water region at 4 °C (5.2–4.9 ppm) was excluded, along with the spectral regions > 9.0 ppm and <0.3 ppm.

Using the Wiley KnowItAll software (Wiley Science Solution, Hoboken, NJ, USA, KnowItAll Metabolomics Edition v17.0.117.0), NMR spectral regions (‘bins’) were defined using the KnowItAll intellibucket™ option. The more obvious resonances were assigned to metabolites of interest on the basis of literature values of chemical shifts [26], and, if necessary, the component bins within a given metabolite region were summed: formate (8.48–8.42 ppm), lipid-C**H**=C**H**- (L7) (5.40–5.27 ppm), glucose doublet (5.26–5.21 ppm), total choline region (tCho) (including trimethylamine-N-oxide, glycerophosphocholine, phosphocholine and choline) (3.29–2.19 ppm), lipid-CH=CHC**H_2_**-CH=CH- (polyunsaturated fatty acids, L6) (2.82–2.72 ppm), acetate (1.94–1.91 ppm), alanine (1.50–1.45 ppm), lactate (1.34–1.32 ppm), (lipid-C**H_2_** (L2) (1.32–1.24 ppm), ethanol (1.21–1.16 ppm), and lipid-C**H_3_** (L1) (0.98–0.84 ppm). Several peaks remain to be assigned, and these have been denoted with an asterisk (*). No additional curve fitting was conducted within each defined metabolite region or within each bin.

Using generated tables of signal intensities according to the binned regions, unsupervised multivariate analyses (principal component analysis, PCA) were undertaken using SIMCA 17.0 (Sartorius). To investigate inter-subject variability, ‘patient-averaged’ data were generated from each spectral region/bin, and this dataset was then used for PCA. Patient-specific averages were also used to adjust pairs of matched tumour and surrounding tissue samples originating from the same patient, and these ‘patient-adjusted’ data were then used to investigate differences between tumour and surrounding tissue by unsupervised PCA. Paired statistical analyses of signal levels from a subset of metabolites (formate, glucose, total choline region, acetate, and lipids), unadjusted for the mass of tissue loaded in the disposable insert, from the tumour and the surrounding region were undertaken in GraphPad Prism (Boston, MA, USA) v9.5.1 using the non-parametric Wilcoxon matched-pairs signed-rank test.

### 4.2. Histology

N = 10 HCC tumour and surrounding snap-frozen tissue samples were thawed and fixed in 10% neutral buffered formalin (NBF) overnight at 4 °C. The slices were transferred into cassettes and processed using a Leica TP1020 automated paraffin tissue processor by passing them through ascending degrees of ethanol (EtOH) from 70% to 95% to 100% for 1.5 h each, followed by maintaining in 100% ethanol for 2.5 h. The slices were then clarified using xylene for 3 h to allow for the impregnation of paraffin (PFM medical) for 3 h into the tissue at 65 °C. Individual pieces were transferred to a paraffin-based embedder Bio-Optica BEC150, which was warmed up to 60–75 °C. The tissues were orientated in a mould and allowed to set in paraffin at room temperature (RT). The paraffin blocks were then cooled and sectioned at a thickness of 4 μm using a Rotary 3003 PFM microtome and transferred onto glass slides. Slides were air-dried overnight at RT before being stored.

The deparaffinisation process started by placing the slides containing 4 μm-thick sections in an oven to melt the paraffin at 60 °C for 1 h. The slides were then incubated in xylene twice (10 min each) and rehydrated in descending levels of 100%, 96%, and 70% ethanol (3 min each) and dH_2_O (5 min). The slides were then stained with Harris Haematoxylin (8 min) followed by rinsing in warm running tap water (3 min). Next, the slides were briefly differentiated in 1% acid alcohol (1% HCL in EtOH, 1–2 dips maximum) and running tap water (1–2 min) and counterstained with Eosin (5–6 min). Following a final wash in running water (2–3 min), the slides were dehydrated using ascending grades of EtOH (70%, 90%, and 100% EtOH for 1 min each). In a chemical safety cabinet, the slides were washed in xylene twice (2 min each). Lastly, a coverslip was mounted using DPX (Sigma-Aldrich 44581, Gillingham, UK) and slides were left to dry overnight. The slides were imaged using brightfield microscopy (Olympus BX43 Microscope, Olympus, Tokyo, Japan).

## 5. Conclusions

In conclusion, given the complexity of HCC tumours, a nuanced overview of documenting tissue metabolism is relevant. Quantifying the metabolic intricacy involves understanding altered glucose metabolism and lipid signalling, as well as variations in low molecular weight metabolite pathways, all of which play a pivotal role in tumour sustenance and progression. Understanding these metabolic differences not only provides insights into the molecular features of HCC but may also suggest potential metabolic targets suitable for therapeutic approaches. The ability to confirm and extend existing diagnostic procedures, at least in terms of histopathological assessments, may validate the use of HRMAS-NMR techniques to help diagnostic decisions and treatments where more clarity on metabolic aspects of HCC would be beneficial.

## Figures and Tables

**Table 1:** Clinical details of the N = 10 study cohort. Abbreviations: N/A, not available; BMI, body mass index; BCLC stage, Barcelona Clinic Liver Cancer stage; T2DM, Type 2 diabetes mellitus.

Sample Details		Clinical Details								Tumour Characteristics					Background Liver Tissue to Tumour	
ID	Aetiology from Histology	Age yr	BMI	Ethnicity	Sex M/F	BCLC Stage	Alcohol Units per wk	T2DM	Other Medication	Primary Tumour Size cm	Tumour Multiplicity	Vascular Invasion	Tumour Steatosis %	Tumour Differentiation	Background Liver Tissue Fibrosis	Background Liver Tissue Steatosis %
039	Alcohol	70	29.3	Caucasian	M	A	None	No	N/A	2.7	Single	Y	0	Moderate	6 (cirrhosis)	70
141	SLD + alcohol	82	UA	Caucasian	M	N/A	N/A	No	N/A	8.0	N/A	Y	0	Moderate	1	20
116	SLD	78	37.7	Caucasian	F	A	None	Yes	N/A	5.5	Single	y	<5	Moderate	1	20
134	SLD	69	35.6	Caucasian	M	A	9	Yes	Atrovastatin, Bendroflumethiazide, Glicazide, Omeprazole, Perindopril, Tamsulosin, Sodium alginate, TDS	3.6	Single	N	Mostly necrotic	Mostly necrotic	6 (cirrhosis)	20
168	Not discernible (SLD clinically)	70	22.8	Caucasian	M	A	6	Yes	Metformin, Empagliflozin, Tamsulosin, Atoravstatin	15.5	Single	Y	0	Poor	1	<5
117	Not discernible (subsequent SLD ^a^)	88	32.7	Caucasian	M	N/A	4	No	Ramipril, Apixaban, Simvastatin, Amlopidine, Furosemide, Bisoprolol, Tamsulosin	8.0	Single	N	40	Moderate	1	0
107	Not discernible	66	16.7	Caucasian	F	N/A	None	No	N/A	2.0	Single	N/A	<5	Poor	1	0
235	Not discernible	77	25.8	Caucasian	F	B	Social only	Yes	N/A	1.3	Multiple	N	0	Moderate	1	0
268	Not discernible	53	20.0	Caucasian	F	N/A	N/A	No	N/A	4.9	Multiple	Y	<5	Moderate	1	<5
099	Treated HCV	61	20.8	Caucasian	F	B	10	No	Amitriptyline, Simvastatin, Zopiclone, Sertraline, Salbutamol inhaler, Co-codamol, Spiriva inhaler	5.4	Multiple	Y	0	Moderate	3	0

^a^ A diagnosis of SLD was made five years after surgery.

## Data Availability

The datasets generated during and/or analysed during the current study are available from the corresponding author upon reasonable request.

## Supplementary Materials

The following supporting information can be downloaded at: https://www.mdpi.com/article/10.3390/ijms25168924/s1.

## Abbreviations

Ace, acetate; Ala, alanine; BMI, body mass index; CLD, chronic liver disease; CPMG, Carr-Purcell-Meiboom-Gill; FMT, faecal microbiota transplant; For, formate; Glc, glucose; HBV, hepatitis-B virus; HCC, hepatocellular carcinoma; HCV, hepatitis-C virus; HRMAS, high-resolution magic angle spinning; ID, identifier; Kel-F^®^, polychlorotrifluoroethylene; L1, lipid-C**H_3_**; L2, lipid-C**H_2_**-; L3, lipid C**H_2_**CH_2_CO; L4, lipid CH=CHC**H_2_**CH_2_-; L5, lipid CH_2_C**H_2_**CO; L6, lipid-CH=CHC**H_2_**-CH=CH-; L7, lipid-C**H**=C**H**-; Lac, lactate; MASLD, metabolic dysfunction-associated steatotic liver disease; NBF, neutral buffered formalin; PCTFE, polychlorotrifluoroethylene; PD-1, programmed cell death protein 1; PCA, principal component analysis; Phe, phenylalanine; RT, room temperature; SLD, steatotic liver disease; T2DM, Type 2 diabetes mellitus; tCho, total choline-containing compounds; Tyr, tyrosine.

## References

[1] Yang J.D., Hainaut P., Gores G.J., Amadou A., Plymoth A., Roberts L.R. (2019). A global view of hepatocellular carcinoma: Trends, risk, prevention and management. Nat. Rev. Gastroenterol. Hepatol..

[2] Forner A., Reig M., Bruix J. (2018). Hepatocellular carcinoma. Lancet.

[3] Calderon-Martinez E., Landazuri-Navas S., Vilchez E., Cantu-Hernandez R., Mosquera-Moscoso J., Encalada S., Al Lami Z., Zevallos-Delgado C., Cinicola J. (2023). Prognostic Scores and Survival Rates by Etiology of Hepatocellular Carcinoma: A Review. J. Clin. Med. Res..

[4] Younossi Z.M., Wong G., Anstee Q.M., Henry L. (2023). The Global Burden of Liver Disease. Clin. Gastroenterol. Hepatol..

[5] Rumgay H., Arnold M., Ferlay J., Lesi O., Cabasag C.J., Vignat J., Laversanne M., McGlynn K.A., Soerjomataram I. (2022). Global burden of primary liver cancer in 2020 and predictions to 2040. J. Hepatol..

[6] Burton A., Balachandrakumar V.K., Driver R.J., Tataru D., Paley L., Marshall A., Alexander G., Rowe I.A., Palmer D.H., Cross T.J.S. (2022). Regional variations in hepatocellular carcinoma incidence, routes to diagnosis, treatment and survival in England. Br. J. Cancer.

[7] Kaffe E., Tisi A., Magkrioti C., Aidinis V., Mehal W.Z., Flavell R.A., Maccarrone M. (2023). Bioactive signalling lipids as drivers of chronic liver diseases. J. Hepatol..

[8] Tilg H., Adolph T.E., Dudek M., Knolle P. (2021). Non-alcoholic fatty liver disease: The interplay between metabolism, microbes and immunity. Nat. Metab..

[9] Cheng X., Wang W., Zhang Z., Zhang H., Zhu P., He R., Wu M., Zhou T., Jiang Y., Jiang L. (2023). Distinctly altered lipid components in hepatocellular carcinoma relate to impaired T cell-dependent antitumor immunity. Hepatol. Int..

[10] Riva A., Gray E.H., Azarian S., Zamalloa A., McPhail M.J.W., Vincent R.P., Williams R., Chokshi S., Patel V.C., Edwards L.A. (2020). Faecal cytokine profiling as a marker of intestinal inflammation in acutely decompensated cirrhosis. JHEP Rep..

[11] Sharma L., Riva A. (2020). Intestinal Barrier Function in Health and Disease—Any Role of SARS-CoV-2?. Microorganisms.

[12] Li M., van Esch B.C.A.M., Wagenaar G.T.M., Garssen J., Folkerts G., Henricks P.A.J. (2018). Pro- and anti-inflammatory effects of short chain fatty acids on immune and endothelial cells. Eur. J. Pharmacol..

[13] King R.J., Singh P.K., Mehla K. (2022). The cholesterol pathway: Impact on immunity and cancer. Trends Immunol..

[14] Zhao J., Lee K., Toh H.C., Lam K.P., Neo S.Y. (2023). Unravelling the role of obesity and lipids during tumor progression. Front. Pharmacol..

[15] Montironi C., Castet F., Haber P.K., Pinyol R., Torres-Martin M., Torrens L., Mesropian A., Wang H., Puigvehi M., Maeda M. (2023). Inflamed and non-inflamed classes of HCC: A revised immunogenomic classification. Gut.

[16] Llovet J.M., Castet F., Heikenwalder M., Maini M.K., Mazzaferro V., Pinato D.J., Pikarsky E., Zhu A.X., Finn R.S. (2022). Immunotherapies for hepatocellular carcinoma. Nat. Rev. Clin. Oncol..

[17] Ohtani N., Kamiya T., Kawada N. (2023). Recent updates on the role of the gut-liver axis in the pathogenesis of NAFLD/NASH, HCC, and beyond. Hepatol. Commun..

[18] Routy B., Le Chatelier E., DeRosa L., Duong C.P.M., Alou M.T., Daillère R., Fluckiger A., Messaoudene M., Rauber C., Roberti M.P. (2018). Gut microbiome influences efficacy of PD-1–based immunotherapy against epithelial tumors. Science.

[19] Matson V., Fessler J., Bao R., Chongsuwat T., Zha Y., Alegre M.-L., Luke J.J., Gajewski T.F. (2018). The commensal microbiome is associated with anti–PD-1 efficacy in metastatic melanoma patients. Science.

[20] Gopalakrishnan V., Spencer C.N., Nezi L., Reuben A., Andrews M.C., Karpinets T.V., Prieto P.A., Vicente D., Hoffman K., Wei S.C. (2018). Gut microbiome modulates response to anti–PD-1 immunotherapy in melanoma patients. Science.

[21] Komiyama S., Yamada T., Takemura N., Kokudo N., Hase K., Kawamura Y.I. (2021). Profiling of tumour-associated microbiota in human hepatocellular carcinoma. Sci. Rep..

[22] Beckonert O., Coen M., Keun H.C., Wang Y., Ebbels T.M.D., Holmes E., Lindon J.C., Nicholson J.K. (2010). High-resolution magic-angle-spinning NMR spectroscopy for metabolic profiling of intact tissues. Nat. Protoc..

[23] Cheng L.L. (2023). High-resolution magic angle spinning NMR for intact biological specimen analysis: Initial discovery, recent developments, and future directions. NMR Biomed..

[24] Schenetti L., Mucci A., Parenti F., Cagnoli R., Righi V., Tosi M.R., Tugnoli V. (2006). HR-MAS NMR spectroscopy in the characterization of human tissues: Application to healthy gastric mucosa. Concepts Magn. Reson. Part A.

[25] Faitot F., Besch C., Battini S., Ruhland E., Onea M., Addeo P., Woehl-Jaeglé M.-L., Ellero B., Bachellier P., Namer I.-J. (2018). Impact of real-time metabolomics in liver transplantation: Graft evaluation and donor-recipient matching. J. Hepatol..

[26] Duarte I.F., Stanley E.G., Holmes E., Lindon J.C., Gil A.M., Tang H., Ferdinand R., McKee C.G., Nicholson J.K., Vilca-Melendez H. (2005). Metabolic assessment of human liver transplants from biopsy samples at the donor and recipient stages using high-resolution magic angle spinning 1H NMR spectroscopy. Anal. Chem..

[27] Martínez-Granados B., Morales J.M., Rodrigo J.M., Del Olmo J., Serra M.A., Ferrández A., Celda B., Monleón D. (2011). Metabolic profile of chronic liver disease by NMR spectroscopy of human biopsies. Int. J. Mol. Med..

[28] Yang Y., Li C., Nie X., Feng X., Chen W., Yue Y., Tang H., Deng F. (2007). Metabonomic studies of human hepatocellular carcinoma using high-resolution magic-angle spinning 1H NMR spectroscopy in conjunction with multivariate data analysis. J. Proteome Res..

[29] Solinas A., Chessa M., Culeddu N., Porcu M.C., Virgilio G., Arcadu F., Deplano A., Cossu S., Scanu D., Migaleddu V. (2014). High resolution-magic angle spinning (HR-MAS) NMR-based metabolomic fingerprinting of early and recurrent hepatocellular carcinoma. Metabolomics.

[30] Cao D., Cai C., Ye M., Gong J., Wang M., Li J., Gong J. (2017). Differential metabonomic profiles of primary hepatocellular carcinoma tumors from alcoholic liver disease, HBV-infected, and HCV-infected cirrhotic patients. Oncotarget.

[31] Cacciatore S., Hu X., Viertler C., Kap M., Bernhardt G.A., Mischinger H.-J., Riegman P., Zatloukal K., Luchinat C., Turano P. (2013). Effects of Intra- and Post-Operative Ischemia on the Metabolic Profile of Clinical Liver Tissue Specimens Monitored by NMR. J. Proteome Res..

[32] Ter Voert E.E.G.W., Heijmen L., van Asten J.J.A., Wright A.J., Nagtegaal I.D., Punt C.J., de Wilt J.H., van Laarhoven H.W., Heerschap A. (2019). Levels of choline-containing compounds in normal liver and liver metastases of colorectal cancer as recorded by ^1^H MRS. NMR Biomed..

[33] Imperiale A., Poncet G., Addeo P., Ruhland E., Roche C., Battini S., Cicek A.E., Chenard M.P., Hervieu V., Goichot B. (2019). Metabolomics of Small Intestine Neuroendocrine Tumors and Related Hepatic Metastases. Metabolites.

[34] Rivas M.P., Aguiar T.F.M., Maschietto M., Lemes R.B., Caires-Júnior L.C., Goulart E., Telles-Silva K.A., Novak E., Cristofani L.M., Odone V. (2020). Hepatoblastomas exhibit marked *NNMT* downregulation driven by promoter DNA hypermethylation. Tumor Biol..

[35] Tasic L., Avramović N., Jadranin M., Quintero M., Stanisic D., Martins L.G., Costa T.B.B.C., Novak E., Odone V., Rivas M. (2022). High-Resolution Magic-Angle-Spinning NMR in Revealing Hepatoblastoma Hallmarks. Biomedicines.

[36] Cobbold J.F.L., Cox I.J., Brown A.S., Williams H.R.T., Goldin R.D., Thomas H.C., Thursz M.R., Taylor-Robinson S.D. (2012). Lipid profiling of pre-treatment liver biopsy tissue predicts sustained virological response in patients with chronic hepatitis C. Hepatol. Res..

[37] Nachabé R., van der Hoorn J.W.A., van de Molengraaf R., Lamerichs R., Pikkemaat J., Sio C.F., Hendriks B.H.W., Sterenborg H.J.C.M. (2012). Validation of interventional fiber optic spectroscopy with MR spectroscopy, MAS-NMR spectroscopy, high-performance thin-layer chromatography, and histopathology for accurate hepatic fat quantification. Investig. Radiol..

[38] Peeters F., Cappuyns S., Piqué-Gili M., Phillips G., Verslype C., Lambrechts D., Dekervel J. (2024). Applications of single-cell multi-omics in liver cancer. JHEP Rep..

[39] Jagatia R., Doornebal E.J., Rastovic U., Harris N., Feyide M., Lyons A.M., Miquel R., Zen Y., Zamalloa A., Malik F. (2023). Patient-derived precision cut tissue slices from primary liver cancer as a potential platform for preclinical drug testing. EBioMedicine.

[40] Fernandes W., Harris N., Adofina L., Zamalloa A., Heaton N., Menon K., Srinivasan P., Miquel R., Zen Y., Kelly G. (2024). WED-542-YI Unravelling bacterial metabolites using high-resolution magic angle spinning nuclear magnetic resonance spectroscopy in intact hepatocellular carcinoma liver tissue. J. Hepatol..

[41] Rockey D.C., Caldwell S.H., Goodman Z.D., Nelson R.C., Smith A.D. (2009). Liver biopsy. Hepatology.

[42] Renault M., Shintu L., Piotto M., Caldarelli S. (2013). Slow-spinning low-sideband HR-MAS NMR spectroscopy: Delicate analysis of biological samples. Sci. Rep..

[43] André M., Dumez J.N., Rezig L., Shintu L., Piotto M., Caldarelli S. (2014). Complete Protocol for Slow-Spinning High-Resolution Magic-Angle Spinning NMR Analysis of Fragile Tissues. Anal. Chem..

[44] Taylor J.L., Wu C., Cory D., Gonzalez R.G., Bielecki A., Cheng L.L. (2003). High-resolution magic angle spinning proton NMR analysis of human prostate tissue with slow spinning rates. Magn. Reson. Med..

[45] Warburg O. (1925). The Metabolism of Carcinoma Cells. J. Cancer Res..

[46] Parhofer K.G. (2015). Interaction between Glucose and Lipid Metabolism: More than Diabetic Dyslipidemia. Diabetes Metab. J..

[47] Weickert M.O., Pfeiffer A.F.H. (2006). Signalling mechanisms linking hepatic glucose and lipid metabolism. Diabetologia.

[48] Rowe J.H., Elia I., Shahid O., Gaudiano E.F., Sifnugel N.E., Johnson S., Reynolds A.G., Fung M.E., Joshi S., LaFleur M.W. (2023). Formate Supplementation Enhances Antitumor CD8+ T-cell Fitness and Efficacy of PD-1 Blockade. Cancer Discov..

[49] Liu Z., Oyetunde T., Hollinshead W.D., Hermanns A., Tang Y.J., Liao W., Liu Y. (2017). Exploring eukaryotic formate metabolisms to enhance microbial growth and lipid accumulation. Biotechnol. Biofuels.

[50] Ternes D., Tsenkova M., Pozdeev V.I., Meyers M., Koncina E., Atatri S., Schmitz M., Karta J., Schmoetten M., Heinken A. (2022). The gut microbial metabolite formate exacerbates colorectal cancer progression. Nat. Metab..

[51] Abbott M., Ustoyev Y. (2019). Cancer and the Immune System: The History and Background of Immunotherapy. Semin. Oncol. Nurs..

[52] Meiser J., Schuster A., Pietzke M., Voorde J.V., Athineos D., Oizel K., Burgos-Barragan G., Wit N., Dhayade S., Morton J.P. (2018). Increased formate overflow is a hallmark of oxidative cancer. Nat. Commun..

[53] Pietzke M., Meiser J., Vazquez A. (2020). Formate metabolism in health and disease. Mol. Metab..

[54] Zheng C., Lu F., Chen B., Yang J., Yu H., Wang D., Xie H., Chen K., Xie Y., Li J. (2023). Gut microbiome as a biomarker for predicting early recurrence of HBV-related hepatocellular carcinoma. Cancer Sci..

[55] Yuan J., Chen C., Cui J., Lu J., Yan C., Wei X., Zhao X., Li N., Li S., Xue G. (2019). Fatty Liver Disease Caused by High-Alcohol-Producing Klebsiella pneumoniae. Cell Metab..

[56] Mbaye B., Wasfy R.M., Alou M.T., Borentain P., Andrieu C., Caputo A., Raoult D., Gerolami R., Million M. (2023). Limosilactobacillus fermentum, Lactococcus lactis and Thomasclavelia ramosa are enriched and Methanobrevibacter smithii is depleted in patients with non-alcoholic steatohepatitis. Microb. Pathog..

