# Supplementary material for: High-Resolution Magic Angle Spinning Nuclear Magnetic Resonance Spectroscopy of Paired Clinical Liver Tissue Samples from Hepatocellular Cancer and Surrounding Region
Source: Int J Mol Sci. 2024 Aug 16;25(16):8924. doi: 10.3390/ijms25168924 (PMC11354908; doi:10.3390/ijms25168924)
Supplement: Supplementary file 1 [file ijms-25-08924-s001.zip › ijms-3102949-supplementary.pdf]

International Journal of *Molecular Sciences*

MDPI

Supplementary Material

High-Resolution Magic Angle Spinning Nuclear Magnetic Resonance Spectroscopy of Paired Clinical Liver Tissue Samples from Hepatocellular Cancer and Surrounding Region

Wendy M. Fernandes [1,2], Nicola Harris [1,2], Ane Zamalloa [3], Lissette Adofina [3], Parthi Srinivasan [3], Krishna Menon [3], Nigel Heaton [3], Rosa Miquel [4], Yoh Zen [4], Geoff Kelly [5], James A. Jarvis [6], Alain Oregioni [5], Shilpa Chokshi [1,2], Antonio Riva [1,2] and I. Jane Cox [1,2,*]

1 The Roger Williams Institute of Hepatology, Foundation for Liver Research, 111 Coldharbour Lane, London SE5 9NT, UK; a.riva@researchinliver.org.uk (A.R.)
2 Faculty of Life Sciences & Medicine, King's College London, London WC2R 2LS, UK
3 Institute of Liver Studies, King's College Hospital NHS Foundation Trust, Denmark Hill, London SE5 9RS, UK
4 Liver Histopathology Laboratory, Institute of Liver Studies, King's College Hospital NHS Foundation Trust, Denmark Hill, London SE5 9RS, UK
5 MRC Biomedical NMR Centre, The Francis Crick Institute, 1 Midland Road, London NW1 1AT, UK
6 Randall Centre for Cell & Molecular Biophysics and Centre for Biomolecular Spectroscopy, King's College London, London SE1 1UL, UK
* Correspondence: j.cox@researchinliver.org.uk; Tel.: +44-(0)20-7255-9830

Table S1. NMR Peak Assignments.

**Peak assignment**	**Chemical shift/ppm**	**Upper bin limit /ppm**	**Lower bin limit /ppm**
Lipid C**H3** (L1)	0.90	0.98	0.84
Lipid C**H2** (L2)	1.20	1.32	1.24
Lipid C**H2**CH2CO (L3)	1.60	1.62	1.55
Lipid CH=CHC**H2**CH2 (L4)	2.04	2.10	1.98
Lipid CH2C**H2**CO (L5)	2.26	2.30	2.22
Lipid CH=CHC**H2**CH=CH (L6)	2.77	2.82	2.72
Lipid C**H**=C**H** (L7)	5.33	5.40	5.27
Valine doublet	1.04	1.06	1.02
Ethanol triplet	1.18	1.21	1.16
Lactate doublet	1.33	1.34	1.32
Alanine doublet	1.48	1.5	1.45
Acetate singlet	1.92	1.94	1.91
Choline	3.20	3.22	3.19
Phosphocholine	3.22	3.25	3.22
TMAO/glycerophosphocholine	3.26	3.29	3.25
Glucose doublet	5.22	5.26	5.21
Histidine	7.10	7.05	6.95
Histidine	7.75	7.80	7.70
Phenylalanine	7.32	7.33	7.28
Phenylalanine	7.34	7.38	7.33
Phenylalanine	7.42	7.44	7.38
Tyrosine	6.85	6.91	6.82
Tyrosine	7.17	7.20	7.13
Formate	8.46	8.48	8.43

Table S2. Summary of selected lipid and low molecular weight metabolite levels in surrounding tissue (sample IDs xxxA) and HCC (sample IDs xxxB) for each subject.

**Sample details**			**Metabolite of interest, including spectral region used for binning definition**								
**ID**	**Aetiology on histology**	**Formate 8.48-8.42ppm**	**Glucose 5.26-5.21ppm**	**3.5-4.0 ppm region**	**Choline region 3.29-3.19ppm**	**Acetate 1.94-1.91ppm**	**Alanine 1.50-1.45ppm**	**L7 5.40-5.27ppm**	**L6 2.82-2.72ppm**	**Lipid- CH_2 1.32-1.24ppm**	**Lipid-CH_3 0.98-0.84ppm**
039A	Alcohol	4.57E+05	4.19E+07	7.66E+08	1.08E+08	1.34E+07	4.47E+07	2.69E+08	9.38E+07	3.37E+09	1.47E+09
039B	Alcohol	5.12E+05	7.94E+06	2.29E+08	9.35E+07	4.55E+06	1.54E+07	2.24E+07	1.27E+07	1.22E+08	6.90E+07
141A	SLD+alcohol	3.63E+05	1.66E+07	1.07E+09	9.32E+07	7.93E+06	4.70E+07	1.33E+08	5.54E+07	1.41E+09	6.53E+08
141B	SLD+alcohol	9.48E+05	1.99E+07	1.37E+09	1.62E+08	1.28E+07	4.76E+07	1.96E+07	1.03E+07	2.29E+08	1.43E+08
116A	SLD	1.54E+06	6.22E+07	1.18E+09	3.60E+08	1.75E+07	8.37E+07	2.47E+08	1.02E+08	3.45E+09	1.55E+09
116B	SLD	1.11E+06	6.19E+07	1.42E+09	4.46E+08	1.24E+07	6.53E+07	6.61E+07	2.03E+07	1.10E+09	5.05E+08
134A	SLD	5.58E+05	6.03E+07	7.23E+08	2.70E+08	1.69E+07	7.86E+07	3.32E+08	1.27E+08	4.68E+09	2.03E+09
134B	SLD	6.01E+05	3.72E+07	1.26E+08	3.27E+07	1.39E+07	5.22E+07	3.63E+08	6.28E+07	6.03E+09	2.67E+09
168A	SLD	4.02E+05	1.73E+07	6.14E+08	2.00E+08	9.25E+06	4.13E+07	8.62E+07	4.01E+07	1.02E+09	4.23E+08
168B	SLD	1.18E+06	6.74E+06	3.22E+08	1.10E+08	1.12E+07	3.58E+07	9.52E+06	1.07E+07	7.42E+07	8.38E+07
117A	None→ SLD	4.08E+05	2.10E+07	5.34E+08	2.20E+08	8.62E+06	3.98E+07	5.04E+07	2.68E+07	5.71E+08	2.63E+08
117B	None→ SLD	5.87E+05	5.19E+07	5.86E+08	2.62E+08	1.56E+07	7.46E+07	3.11E+08	9.10E+07	4.32E+09	1.89E+09
107A	None	4.25E+05	1.41E+07	1.00E+09	2.22E+08	6.97E+06	1.77E+07	1.63E+07	9.38E+06	1.41E+08	8.08E+07
107B	None	7.19E+05	4.36E+07	1.67E+09	2.68E+08	8.79E+06	2.67E+07	2.10E+07	1.33E+07	1.38E+08	8.22E+07
235A	None	8.29E+05	2.18E+07	1.11E+09	2.47E+08	1.74E+07	6.75E+07	1.97E+07	1.41E+07	2.13E+08	1.54E+08
235B	None	6.36E+05	2.30E+07	9.66E+08	2.61E+08	1.23E+07	3.66E+07	1.61E+07	1.15E+07	2.29E+08	1.47E+08
268A	None	5.20E+05	2.43E+07	1.08E+09	2.29E+08	1.09E+07	4.39E+07	4.06E+07	2.42E+07	5.42E+08	2.76E+08
268B	None	1.70E+06	3.54E+07	1.29E+09	4.65E+08	7.04E+06	8.02E+07	2.73E+07	1.19E+07	4.42E+08	2.04E+08
099A	Treated HCV	6.17E+04	3.33E+07	1.30E+09	2.25E+08	1.23E+07	4.35E+07	3.85E+06	1.68E+07	1.28E+08	8.52E+07
099B	Treated HCV	5.98E+05	4.26E+06	1.35E+09	8.33E+07	7.52E+06	1.95E+07	1.47E+07	5.27E+06	1.71E+08	9.71E+07
Samples A	Median	4.41E+05	2.31E+07	1.04E+09	2.24E+08	1.16E+07	4.43E+07	6.83E+07	3.35E+07	7.96E+08	3.50E+08
Samples A<br>Samples A	Q1<br>Q3	3.92E+05,<br>6.26E+05	1.71E+07,<br>4.65E+07	6.96E+08,<br>1.13E+09	1.77E+07,<br>2.53E+08	8.45E+06,<br>1.70E+07	4.09E+07,<br>7.03E+07	1.89E+07,<br>2.53E+08	1.61E+07,<br>9.59E+07	1.95E+08,<br>3.39E+09	1.49E+08, 1.37E+09
Samples B	Median	6.78E+05	2.92E+07	1.13E+09	2.12E+08	1.18E+07	4.21E+07	2.17E+07	1.23E+07	2.29E+08	1.43E+08
Samples B<br>Samples B	Q1<br>Q3	5.95E+05,<br>1.13E+06	7.64E+06,<br>4.75E+07	2.99E+08,<br>1.38E+09	9.10E+07,<br>3.13E+08	7.40E+06,<br>1.31E+07	2.49E+07,<br>6.76E+07	1.58E+07,<br>1.27E+08	1.06E+07,<br>3.09E+07	1.34E+08,<br>1.91E+09	8.30E+07,<br>1.20E+09
